# There and back again: A sperm's tale

**DOI:** 10.1002/mrd.23325

**Published:** 2020-02-05

**Authors:** Melissah Rowe, Patrice Rosengrave

**Affiliations:** ^1^ Department of Animal Ecology Netherlands Institute of Ecology (NIOO‐KNAW) Wageningen The Netherlands; ^2^ Department of Pathology and Biomedical Science University of Otago Christchurch New Zealand

**Keywords:** biology of spermatozoa, ejaculate‐female interactions, gamete biology, post‐copulatory sexual selection, reproduction, sperm evolution

In the mid‐20th century, two Swedish biologists—Åke Franzén and Bjorn Afzelius—significantly advanced the study of sperm biology through the investigation of sperm ultrastructure using the newly developed electron microscope. Franzén studied sperm in some 200 (mostly marine) invertebrate species, linking sperm form and structure to the fertilization environment. Most notably, Franzén found that sperm of external fertilizers were relatively small and of the “primitive” type, while animals with internal sperm transfer tended to have more elongated and modified sperm morphologies (Franzén l955, 1956a, 1956b). In similarly ground‐breaking work, Afzelius provided the first documentation of pores in the sperm nucleus membrane, the first high‐magnification image of the acrosome reaction, and discovered the dynein arms on the microtubule doublets of the sperm flagellum, the latter providing clues to the mechanism of sperm motility (Afzelius, 1957, 1959). These findings stimulated research on sperm structure and function, prompted the use of sperm ultrastructure as a phylogenetic and taxonomic tool, and motivated investigations of the role of cilia and flagella in human disease.

Given the striking variation in sperm size and shape observed across the animal kingdom—the sperm cell is now recognized as the most diverse cell type known (Pitnick, Hosken, & Birkhead, 2009)—it is perhaps not surprising that there is still much to learn. Indeed, the desire to understand sperm structure and function that drove Franzén and Afzelius still motivates researchers today. The biology of spermatozoa (BoS) meetings were established in the early 1990's by Tim Birkhead and Harry Moore, both from the University of Sheffield, as a forum to advance knowledge of sperm biology, through the exchange of ideas across a range of disciplines including evolutionary biology, cell physiology, and human reproductive health. As an added bonus, these biennial meetings took place in the beautiful English countryside of the Peak District.

In 2019, BoS moved to the Swedish town of Nynäshamn for the 15th BoS meeting. Under the direction of the international steering committee—comprised of Rhonda Snook (Stockholm University), John Fitzpatrick (Stockholm University), David Hosken (University of Exeter), Scott Pitnick (Syracuse University), Lukas Schärer (University of Basel), and Nina Wedell (University of Exeter)—this new venue was a resounding success. All the things that have made previous BoS meetings so successful were carried over to Sweden. With no concurrent sessions and presentations focused on unpublished and in‐progress research with 15 min allocated to discussion, the meeting provided the perfect environment for the cross‐fertilization of research ideas and discussion of emerging topics and methodologies. BoS15 also continued the tradition of inviting 2–3 speakers whose research offers new perspectives and approaches from other disciplines. An amazing scientific program, combined with a venue that allowed for discussion long into the night and a walk along the beautiful Nynäshamn coast, meant that BoS15 was the perfect blend of the old and the new.

Kicking off the BoS15 scientific program, Tim Birkhead (University of Sheffield) transitioned the 80 BoS delegates from the Peak District of England to the Swedish coastal town of Nynäshamn with the greatest of ease with his opening plenary talk. By first acknowledging the contributions of Franzén and Afzelius to sperm biology, it was almost as if BoS was “back home again.” Birkhead then took us on a stroll down memory lane, reliving the highlights of BoS over the years and reminding attendees of why this meeting is just so good, and the reasons for why we all keep coming back. It has been said by many a BoS attendee, that this meeting is their favorite professional conference. The warm and welcoming environment, provided by the steering committee and attendees, makes this conference feel like a family “catch‐up,” not to mention the high‐quality research presented and thought‐provoking new ideas that keep the study of gamete biology moving forward in new directions. Once bitten by a BoS meeting, there is no turning back.

The 15th BoS meeting merged the old and the new in more ways than one. Following in the footsteps of Frankén and Afzelius, several talks focused on explaining the causes and consequences of sperm variation. Kristin Hook (University of Maryland) combined electron microscopy with computer‐assisted‐sperm‐analysis methods to unravel the functional significance of complex sperm morphology and sperm aggregate behavior, as well as the role female promiscuity plays in the evolution of these traits. Using modern phylogenetic and evolutionary analysis methods, Ariel Kahrl (Stockholm University) shed new light on the role of the fertilisation environment for the evolution of sperm size. Compiling data on more than 3,000 animal species, this work linked evolutionary transitions in fertilisation environment to shifts in both sperm size and the rate of sperm evolution, providing compelling evidence for the evolution of longer sperm, and a higher rate of evolutionary change in sperm size in internally fertilizing taxa compared with those with external fertilisation. Exemplifying the community nature of these BoS meeting, Kahrl et al plan to make this resource publicly available in the future (SpermTree.org). Finally, Sara Calhim (University of Jyvaskyla) reminded us of how much more we have to learn about sperm variation and the benefit of studying “novel” animal groups by introducing to us the weird and wonderful world of tardigrade sperm. Sperm with no apparent mitochondria or floating mitochondria. Sperm with baton, spiral, or filamentous head shapes. Not to mention the morphological changes sperm undergo when they are inside the female versus in the male testis. Importantly, anyone can contribute to this work by sending samples of moss (the home to these little water bears) to Calhim. Check out how to do that here: https://tinyurl.com/sendusyourmoss.

Like many BoS meetings before this one, the focus was not entirely on sperm. This was exemplified by the plenary talk “The other gamete: development and evolution of eggs, inside and out” by Cassandra Extavour (Harvard University). Extavour presented an incredible collection of data on egg size and shape variation in insects, where egg volume ranges across eight orders of magnitude, and demonstrated the link between the evolution of egg morphological diversity and ecology (Church, Donoughe, de Medeiros, & Extavour, 2019a). For example, where eggs are laid helps to explain egg variation, with eggs laid in or on water or in the bodies of other animals tending to be smaller, while eggs laid on soil tend to be larger. Reflecting the movement towards open science, Extavour et al have made this database publicly available (Church, Donoughe, de Medeiros, & Extavour, 2019b). Extavour also described how insect embryos are built; detailing years of collaborative research from her lab investigating nuclei movement within the cytoplasm during blastoderm formation and impressing us with videos of nuclei movement captured using long‐term cell tracking. Female diversity was also the topic of Patty Brennan's talk (Mount Holyoke College), who showcased vaginal diversity in dolphins and discussed the potential roles of natural and sexual selection in shaping vaginal morphology. Intriguingly, this variation in vaginal morphology does not appear to be explained by phylogeny, but instead appears likely to reflect the coevolution of male and female genital morphology and may provide a mechanism by which females can regulate copulatory success.

Since the early days of the BoS meetings, researchers have understood the need to better integrate female‐mediated processes into the study of sperm biology and sperm competition (Pitnick & Karr, 1995). The 15th BoS meeting showed how far we have come, with the presented research demonstrating our greater understanding of the dynamic interactions between ejaculates and the female reproductive tract and/or reproductive fluids. We now know that the female reproductive tract provides a selective and interactive environment through which sperm must navigate. Several talks highlighted the role that postmating female–male interactions play on sperm function, female behavior, and, ultimately, reproductive success. These complex interactions were the focus of the first day's plenary talk by Sabine Koelle (University College in Dublin), whose research focuses on reproductive medicine and assisted reproductive technologies in humans. Koelle wowed us all with her videos of sperm swimming within the female reproductive tract under near “in vivo” conditions, which were obtained using a newly developed technique—probe‐based confocal laser endomicroscopy. Koelle's work evoked a great deal of discussion throughout the meeting. This work highlighted the fact that a sperm's journey to the oocyte may be even more complicated than previously thought, questioning the importance of sperm motility versus female reproductive tract contractions for sperm transport. Additionally, Koelle's work suggested fertilization success may be more dependent upon an intact sperm membrane, rather than normal sperm morphology. This is because damage to the membrane impedes the ability of sperm to locate the oocyte. Therefore assessing sperm membrane integrity may be an important assessment tool when investigating idiopathic infertility for humans and animals. Also highlighting the importance of male–female interactions, Emma Whittington (Syracuse University) described how the *Drosophila melanogaster* sperm proteome changes after being transferred to the female. Whittington provided a framework for thinking about the interactive role the female reproductive tract and fluids play in post‐ejaculatory modifications to sperm. Next, Yasir Ahmed‐Braimah (Syracuse University), created a vibrant discussion about postmating immune responses. Ahmed‐Braimah demonstrated that mating triggers immune responses in the female reproductive tract, with ejaculates from heterospecific males mounting a greater immune response than conspecific ejaculates.

Nathan Clark's plenary talk (University of Utah) showed us that male–female interactions in the cabbage white butterfly can be a veritable “battle of the sexes.” In this system, males deposit a large spermatophore, which consists of a virtually indestructible hard outer shell with a soft nutritious inner shell surrounding a ball of sperm, in the female reproductive tract (bursa copulatrix). The spermatophore's nutritious inner shell acts as a nuptial gift. However, because the female cannot mate when the bursa copulatrix is filled and it takes 3 days for the female to break down the hard outer shell of the spermatophore, the spermatophore functions to prevent female remating. To combat this, female butterflies produce proteases in the bursa copulatrix which help to chemically digest the spermatophore and, even more impressively, the bursa copulatrix contains an organ called the signum, “a tooth‐like structure” which mechanically chews through the spermatophore's outer shell. To top it off, Clark demonstrated that both the female proteases and male spermatophore proteins are rapidly evolving. Altogether, this was an amazing story of sexual conflict and cooperation.

Male–female interactions are also critical in external fertilizers. Neil Gemmell (University of Otago) highlighted the importance of female–male interactions as the ovarian fluid, surrounding the unfertilized ova, contains proteins that can speed up or slow down the sperm from “hooknose” male chinook salmon. Similarly, using a broadcast spawner, the blue mussel, Jon Evans (University of Western Australia) showed that female derived water surrounding the eggs, can also hinder or enhance the sperm function. Egg water acts as a chemoattract to guide sperm, but also acts to select against extreme combinations of sperm length and sperm swimming speed.

Sperm function and performance can also be influenced by seminal fluid, setting the stage for complex male–male interactions within the female reproductive tract. This was beautifully demonstrated by Mariana Wolfner (Cornell University), who intrigued us with her research on *Drosophila* seminal fluid and the idea of copulation complementation. It seems that *Drosophila* males exploit seminal fluid sex‐peptides produced by rival males, with the second male to mate effectively “rescuing” the first male's fertility. Stefan Lüpold (University of Zurich) moved the conversation forward again by elegantly demonstrating the complexities of male–male–female interactions and highlighting the multivariate nature of selection on sperm form and function.

The environment also impacts reproduction, and several talks on the final afternoon focused on the consequences of changes in the thermal environment for gamete biology and fertility or the impact of social environment on reproductive plasticity. Ramakrishnan Vasudeva (University of East Anglia) showed that temperature increases through adult development resulted in males producing shorter sperm and females producing larger eggs. Moreover, Vasudeva found that this gamete plasticity is adaptive, with pairs showing greater reproductive performance when males and females were exposed to matching thermal conditions. Next up, Benjamin Walsh (University of Liverpool) introduced the concept of “thermal fertility limits” (i.e., the level and duration of thermal stress that renders individuals unable to reproduce; Walsh et al., 2019). Walsh reviewed empirical evidence for the effects of elevated temperatures on reproduction and fertility and recommended the use of standardized approaches to measuring thermal fertility limits. Given that rising global temperatures and increases in the frequency and duration of heatwave events are threatening biodiversity on a global scale, this work is timely and impactful. Finally, the presentation sessions were wrapped up by Suzanne Alonzo (University of California Santa Cruz) who reminded us of the “social side” of sperm competition. Alzono discussed how social and gametic traits in the ocellated wrasse, whereby males exhibit three alternative male reproductive types, interact to help us understand the dynamics of sexual selection. In this species, male types differ in sperm quantity and quality; the next big question is whether they also differ in cognitive function.

As in previous years, the poster sessions were a critical component of BoS15. Kicking of each of the two posters sessions, presenters “advertised” their posters with a 1‐min research pitch that infused a generous dose of fun into the sessions, and John Fitzpatrick was poised nearby with the timer to catch out anyone who went over the 1‐min mark. The posters showcased a diversity of high‐quality research covering a broad range of topics, including cognitive mechanisms of sperm allocation in junglefowl (Yunke Wang, University of Oxford), environmental effects on sperm gene expression (Rowan Lymbery, University of Western Australia), and sperm adaptation to microbes (Oliver Otti, University of Bayreuth). The female side of reproduction was also well‐represented, with posters covering topics such as female reproductive tract protein evolution in *Drosophila* (Caitlin McDonough, Syracuse University) and the role of female body condition on cryptic female choice via ovarian fluid in wrasse (Matthew Kustra, University of California, Santa Cruz). Finally, several of this year's posters highlighted research on postmating prezygotic reproductive barriers in a range of invertebrate systems, including butterflies (Melissa Plakke, University of Kansas), *Drosophila* (Martin Garlovsky, University of Sheffield), and beetles (Erica Larson, University of Denver).

Alas, after three and a half days of cutting‐edge science and rousing discussion, our meeting was over, and a merry group of gamete biologists farewelled each other and commenced their journey home. We are already looking forward to the next meeting, which will again be held in Nynäshamn on September 6–10th, 2021 (https://www.su.se/zoologi/english/research/conferences/welcome‐to‐bos). Though BoS meetings are typically small, with just 60–80 delegates, new attendees are always welcome and encouraged to inject new ideas and enthusiasm for the study of sperm biology. So mark down the dates for the 16th BoS meeting in your calendar now.

## CONFLICT OF INTERESTS

The authors declare that there are no conflict of interests.
